# Fevers of Summer and Winter; Their Causes and Treatment

**Published:** 1849-07

**Authors:** Charles G. Ballard

**Affiliations:** President of the Association


THE NORTH-WESTERN

### MEDICAL AND SURGICAL JOURNAL.

Vol. II.] JULY, 1849. [No. 2,

### Part I.—Original Communications.

### ARTICLE I.

*Fevers of Summer aud Winter—their causes and Treatment.* An address delivered before the Wabash Central Medical Association. By CHARLES G. BALLARD, M. D., President of the Association. Published by order of the Association.

It will suit our purpose on this occasion to divide diseases into those of Summer and of Winter, as we shall endeavor to point out in what they are identical, and in what they differ.

Summer diseases are disorders of secretion, while those of Winter are inflammations of the serous membranes and other parts, superadded to the morbid condition of the secretions, which alone constitutes the Summer diseases.

The heat of Summer predisposes to diseases of secretion by keeping up a stimulation of the capillary vessels, thereby producing a state of debility from constant excitement, by which the equalibrium of circulation is destroyed, and one consequence must necessarily be an altered state of secretion which tends very strongly to the setting up of febrile excitement.

Bilious fever is so common in the great Missisippi Valley, that it attracts our attention more than any, and not unfrequently more than all others put together; and after all that has been said, and columns have been written upon the subject, we are of opinion that it is a disease purely of secretion.

Let us here simply enquire, what do we meet in every examination ot cases of bilious or summer fever? What disorders do we find? And where do we find them? I answer, in the secretions of the skin, kidneys, stomach and liver, and I will add in the circulation, which, by the way we regard as consequent upon, or growing out of the abnormal condition of the secreting organs. These (the morbid condition of the secretions) we think to be the constituent elements, and *all* the constituent elements of bilious fever.

We are aware that a great deal is said about the debility, the irritability, and the morbid condition of the nervous centres, etc., but who I would ask supposes that any impression whether normal or abnormal, pleasurable or painful, is made through any other medium than that of the nerves? Who is wise enough, or metaphysical enough to explain how this is, or what it is? If in the great crucible of nature things are found so sublimated or refined as to elude investigation, we should have nothing to do with them. It is only when they are tangible and made appreciable to our minds or senses that an attempt at useful investigation promises the best reward for our labor, therefore all we can reasonably hope to do, for our own improvement and the benefit of mankind, is to investigate disease after it is so far developed as to be made cognisable to the senses.

*Causes of Bilious Fever.*—Every systematic writer on medicine for the last five hundred years, has attributed it to marsh miasmata.

And what, we would enquire, is this miasma that is capable of producing such potent effects upon the human race? We are gravely told that it is a noxious matter in gaseous form, produced by the decomposition of vegetable matter existing at certain seasons of the year in the atmosphere, ready to be inhaled at every inspiration and to do the bidding of the angel of destruction.

A medical man who denies the operation of this very satisfactory, and most potent cause of Bilious fever in all its grades from simple chill to black vomit, runs the risk of being excommunicated for heresy, and sent to the mad house as a lunatic. But with all these fearful consequences before our eyes, we shall on the present occasion dare to deny this long cherished faith, acknowledge our skepticism, and state a few reasons for our dissention.

And first, the boldest advocates of miasm have failed to prove its existence. All their testimony and arguments, the greatest latitude being given to them, only tend to show it inferentially.

Secondly, we object because we frequently see a great deal of bilious disease early in the season before a miasmatist would hardly claim that it was produced by vegetable decomposition, unless he should happen to have a portion of last year's stock on hand.

Thirdly, because we see the same disease in winter when vegetable decomposition, to any available extent, is wholly precluded.

Fourthly, we object because there is about the same amount of vegetable matter in one year as in another and it some way or other disappears about the same time, and consequently if like causes produce like effects as philosophers of old taught us, it seems to me that the present season would not have been so healthy.

Fifthly, inasmuch as vegetable decomposition must be infinately enhanced after vegetation is killed by frost, reasoning *a priori*, we should be led irresistably to the conclusion that bilious disease would be equally multiplied, but, unfortunately for the advocates of this doctrine the very reverse is true. Therefore, after all the examination and reflection we have been able to bestow upon the subject, aided by all the lights which effort has endeavored to throw upon this subject, we are free to acknowledge that it has all failed to produce conviction in our mind, and consequently we feel constrained to reject the theory as altogether untenable, although it places us numerically in a very large minority. We feel strongly inclined to the opinion, whatever difficulties may be in the way of a full and satisfactory explanation of the manner by which their effects are produced, that considering heat and moisture as the cause is a much more rational and defensible theory of bilious disease, and comports much better with the facts in the case.

It is not hard to conceive that the direct application of these agents might produce predispositions, and alterations in the animal economy inconsistent with a state of health without calling up the intermediate agent of miasm.

We have already said that the heat of summer predisposes to bilious disease by its continued stimulation of the capillary vessels, this no one will deny. The nervous filaments must also suffer from the same cause, and a general weakness must be the inevitable result. The secretory vessels of the dermodal structure are brought directly under the influence of solar heat, and are stimulated to excessive action, which must finally end in debility, and it is not uncommon to see cases of cholera morbus, diarrhœa, nausea and vomiting frequently occuring after a few days of great heat in the early part of summer, which generally passes off with but little derangement in the circulation, and rarely terminates in a febrile paroxysm. But this is not always the case for sometimes a most malignant and distressing form of fever is set up and established by one or other of the above diseases. This shows a close connection between them and fever, and seems pretty clearly to manifest their common origin, and these are generally referred to the heat of the season as their cause.

In some parts of Africa under the burning rays of a tropical sun, the moisture of the atmosphere, we are told, is so great that the dew point is only five degrees below the greatest solar heat. There, immersed in that excessive heat and moisture, mankind are placed on the very verge of human existence. No one's life is safe there, even for a few weeks, except those who are born and raised under these blighting influences.

What effect must such a state of atmosphere, heat, and moisture produce on the animal economy and especially on the skin and its secretions, and that without the aid of miasm! But the objector says, our atmosphere is not so bad as that of Africa—neither are our bilious diseases, but will be found to bear about the same relation to those of that climate, that our atmosphere, in point of heat and moisture, bears to that of Africa, or any other tropical climate.

We suppose healthy perspiration to be a sanative process of nature, or in other words that it is an excretion necessary to be thrown off in order to mantain that equable action necessary to health, and if this be true, we think it will require much more ingenuity to explain the vis medicatrix naturæ, or how it is that nature resists their tendencies to produce disease with so much potency, than it will to account for their being able to produce bilious disease without the aid of the monster miasm.

If heat is capable of producing the excitement and debility of which we have spoken, and a great excess of moisture is added to it, and if a healthy action and a normal secretion of the skin is requisite to health, can it be strange that the enervating effects of so much heat, together with the moisture of the atmosphere obstructing cutaneous secretion—it not being able to take it up by the process of solution when it is already surcharged with water—I say is it strange that derangement of secretion should be produced? We suppose that if the secretion of which we are speaking is at all necessary to health, it is equally important when it is thrown upon the surface, that some agent should be provided by which it should be carried off, or otherwise it would counteract its own ultimate end, and the consequence would be, that this excrementitious portion of the blood would be retained in the circulation, and morbid action to a greater or less extent, necessarily ensue. Nature, always perfect in all her works, has kindly provided the means to accomplish this desirable object. The atmosphere is capable of holding a vast amount of water in solution. When dry it absorbs it with great rapidity, always, however, in a direct ratio with its dryness, therefore it follows that as the air approximates a state of saturation, its powers of rapid solution are proportionally diminished, if so we see no difficulty in tracing diseases of the hot season directly to its legitimate cause, without calling in the aid of miasm, or summoning any other spirit from its rest in the "vasty deep" to aid our infirmities.

From what has already been said you will perceive that we incline to the opinion that the causes of bilious disease are two simple natural agents, acting directly on the skin and thereby stimulating, modifying, debilitating and obstructing it in its sanative efforts to preserve the system in a state of health. Here we think is to be found the first link in the chain of morbid action out of which all others follow as cause and effect. We all know that if irritability is not in a direct ratio with debility, that it is very much enhanced by it, and consequently we may not wonder that after the cutaneous vessels have been submitted to continual solar stimulation for a time incompatible with health, that debility will follow, and if a damp state of the atmosphere prevents in any way, either negatively or positively, the elimination and carrying off this deleterious portion of the blood, whatever its qualities may be, I care not, the effects may be different but the result will be certain, disease must and will follow the retention and circulation of a principle which should be thrown off for the purpose of purifying the blood, Suppose that it is a sedative, in that event the blood will not sufficiently stimulate the heart, and consequently its action will be enfeebled, the blood will not circulate with sufficient force and fullness through the stomach, liver, and kidneys to excite them to healthy secretion, and here will be new links added to the chain of morbid action, to say nothing of the necessity of a full and free circulation through the pulmonary ramifications in order to a full and perfect decarbonization of the blood while passing through the lesser circulation.

The immediate cause of summer and winter disease it seems to me is about the same, only applied under different, if not opposite states of the system. In summer the cause of obstruction is applied to the system when debilitated by solar heat, but in winter the obstructing cause is applied to a sysetm braced up by cold and in vigorous action, and inflammation is superadded to disease of simple secretion and is developed on the serous membranes and other parts prone to inflammatory action—thus we have the same bilious disease in winter, complicated with inflammation, which renders the treatment far more perplexing to the practitioner, and the terminus more doubtful and dangerous to the patient.

As we have neither time, nor inclination in this address to argue this matter much farther, we wish, lest we should be misunderstood, to state explicitly our position. In short it is this, that heat is the great predisposing cause of all our summer fevers, and cutaneous obstruction the existing cause of both summer and winter fevers.

It matters not whether the existing or immediate causes are brought about by solar heat so long applied to the surface and inhaled into the lungs as to produce the obstruction by the direct debility of the secreting and exhalent vessels of the skin; or whether it be produced by humidity of the atmosphere, because the result would be precisely the same—viz., the excrementitious portion of the blood which nature designed to throw off by perspiration, would be retained in the circulation.

It now remains to say but a few words on the causes of death by fever, and close this part of our address with a few very brief general directions on the plan of treatment best calculated to avert a fatal termination. There are but three ways at most by which fever terminates fatally. They are congestion, inflammation, and exhaustion. If engorgements are suffered slowly to take place in the liver, or kidneys, healthy secretions may be suspended a length of time incompatable with life; death also very frequently happens from cerebral venous accumulations. Inflammation may also set up in these or other organs and produce disorganizations, necessarily fatal, or the system may become so exhausted by the violence of the disease as to preclude the possibility of recovery. The latter, however, we are inclined to think too often depends on one, or both the other causes, and we are decidedly of the opinion that if the practitioner treats his case with judgement and discretion, he will have but little cause to fear a fatal termination from debility alone.

The query now arises, is it possible so to treat fever as to avoid inflammation on the one hand and congestion on the other, and to prevent them from rnnning on to such an extent as seriously to endanger the life of the patient? We think after a long experience in this matter, that it is altogether practible in a great majority of cases. And, without attempting to say what the specific treatment should be in an individual case, we say in general terms that it is the buisness of the physician at each and every visit to ascertain what lesions of secretion, circulation, or inflammation exist and meet them promptly with appropriate remedies; and, with a warning voice, we would say do no more; for by goading an organ unnecessarily you not only add to the stock of general debility, but you may establish inflammation or excite congestion ultimately fatal to the patient.

It is the practice of not a few who call themselves physicians when called to a case of fever, to make a prescription something like this—a dose of calomel and ipecacuanha, febrifuge powders, spts. nit., epispastics applied to some part of the subject, and, as it is fashionable to give quinine, this part of the medication will not be omitted Now this may all be very right and proper, provided there are lesions of secretion of the liver, kidneys, and skin, and also of the nerves and circulation which, it is true, are all sometimes present in a protracted fever case. Yet, even in an extreme case of this kind, it admits a doubt in my mind, whether in a practical point of view this is good practice, when the energies of the system are broken down by disease. Nature generally responds promptly if you call on her at but one or two points at a time, but if you are exorbitant in your demands she will do nothing.

Then I would say when you find a torpid liver, act upon it promptly with its most appropriate stimulant. If morbid action in the kidneys, use some one or more of that class of medicines best calculated to call them into action. And here let me observe that the importance of full and free action of these organs, is not I fear as fully appreciated by many physicians as its merits demand.

It is just as necessary to the safety and speedy recovery of the patient, that the secretions of these glands should be kept in or restored to normal action, as that of the liver, and what is true of these secretions is also true of that of the skin, and although it may be impossible for several days to effect or produce sensible perspiration, still this object should not be lost sight of, and to this end the paroxsyms should be lessened and shortened by frequent spongings of the body with cool salt and water, and the administration of cooling acidulated drinks and perspiratives.

Now it seems to me, and experience proves the fact, that if we promptly call on a secreting organ by its appropriate stimulent, and give it sufficient rest after exciting it into action, for there is such a thing as over stimulating an organ and breaking it down and bringing on engorgement or inflammation (the very thing proposed to avoid,) and we will prevent both engorgement and inflammation of that organ, and so of each in turn.

I have tried to make myself understood on this subject, yet I may have failed.

I have labored to mantain that fever is a disease of the secretion, that the remote cause of summer fever is heat, and the exciting cause is obstructed perspiration, that our winter fevers depend on the same exciting cause, and that the difference between them arises from the fact that in the one fever is induced in a patient debilitated by the heat ot summer, and in the other it is set in action in one braced by the invigorating effects of cold, and consequently assumes an inflammatory type. That the successful management of the summer form consists in preventing congestion or inflammation from being established in any organ essential to life, and that this equalibrium of action can only be maintained or restored by a vigilant attention to the secretions, and when there is a departure from the normal standard in any of them, to apply the remedy best calculated to bring it back to a good state of health.

The winter form of fever will in many instances require the use of the lancet, epispastics, and expectorants.

The congestive form of fever which we hear so much about in some sections of this country, while some authors deny the existence of any such disease, requires vigorous and prompt treatment, and although it rarely if ever appears in this parallel of latitude, yet too many of the profession who never saw a case of congestive fever in all their practice, see as they suppose, its drapery in almost every case of fever, whether inflammatory or otherwise, that falls within their scope of operation, and if they are so unfortunate as to lose a patient fi om whatever cause, they immediately invoke curses on congestive fever, and charge it with the murder of their patients.

In this form of fever, which we have seen something of in a state south of this, the congestion is rapid, coming on in a few minutes, not like the slow engorgements of which we have been speaking. It is ushered in with great distress in the præcordial region, difficult respiration, frequent and deep sighing, shrunken features, pain in the back and head, nausea and sometimes vomiting, cold clammy perspiration, great thirst and restlessness, a constant desire for fresh air, and a sensation of internal heat referable to the stomach or lungs, and sometimes both. If frictions, stimulants and heat to the surface, stimulants and alteratives in copious doses are promptly administered by the mouth, reaction will slowly take place; partial at first, but generally pretty full in the end, which gradually abating, ends in a moderate, warm, healthy perspiration and the patient becomes quite comfortable, and had you not known what his condition had been, you might be led to anticipate but very little trouble with him, but unless active and vigorous measures are promptly adopted and carried out, six or twelve hours will reveal to all concerned, the iminent danger to which he is exposed. You may again succeed in getting him through this paroxysm, but if you do not afford him relief and secure him against a third, you will have nothing to hope.

A few more words and I am done. What is congestion? Or more properly, how is it brought about in such a rapid manner as it frequently takes place? Some have referred it to a weakened action of the heart. Now it would seem that if a weak action of the heart was productive of such alarming and fatal results at one time it must be so at all times, therefore in every and all cases of weakened action of the heart we should have rapid and fatal congestions, which every day's experience contradicts.

Again it would appear to us that in a weak action of that organ depending on organic debility the result must be but one series of fatal congestions. How would this state of things be remedied? Why, the advocate of this theory would tell you, stimulate the heart to stronger action and you will unload the venous system. Suppose you do, can you keep it up *ad libitum?* If not when you cease your stimulation, the debility remaining, not to say increased congestions, must again succeed That congestions do take place is beyond all cavil or dispute, but how, is not so clear.

Whether any man will ever be able to demonstrate this matter so clearly and definately as positively to put the question forever at rest is a matter of some doubt.

We have frequently put the query to ourself, when we have met with deep and sudden congestion in the portal circle or brain—How does this local fullness arise? Why does it take place? Surely not from a general, it must be a local cause. For my part I can conceive of but one pathological condition by which quick and deep engorgement can take place, and that is a partial paralysis of the capillary vessels of the organ congested. This, by relaxing the vessels, thereby enlarging their caliber would allow the blood to flow too freely through them, and venous congestion would consequently ensue.

